# High Fat High Cholesterol Diet (Western Diet) Aggravates Atherosclerosis, Hyperglycemia and Renal Failure in Nephrectomized LDL Receptor Knockout Mice: Role of Intestine Derived Lipopolysaccharide

**DOI:** 10.1371/journal.pone.0141109

**Published:** 2015-11-18

**Authors:** Siddhartha S. Ghosh, Samuel Righi, Richard Krieg, Le Kang, Daniel Carl, Jing Wang, H. Davis Massey, Domenic A. Sica, Todd W. B. Gehr, Shobha Ghosh

**Affiliations:** 1 Divisions of Nephrology, Virginia Commonwealth University, Richmond, Virginia, United States of America; 2 Department of Anatomy and Neurobiology, Virginia Commonwealth University, Richmond, Virginia, United States of America; 3 Division of Pulmonary Medicine, Virginia Commonwealth University, Richmond, Virginia, United States of America; 4 Department of Pathology, Virginia Commonwealth University, Richmond, Virginia, United States of America; 5 Department of Biostatistics, Virginia Commonwealth University, Richmond, Virginia, United States of America; University of Louisville, UNITED STATES

## Abstract

A high fat meal, frequently known as western diet (WD), exacerbates atherosclerosis and diabetes. Both these diseases are frequently associated with renal failure. Recent studies have shown that lipopolysaccharide (LPS) leaks into the circulation from the intestine in the setting of renal failure and after WD. However, it is not clear how renal function and associated disorders are affected by LPS. This study demonstrates that circulatory LPS exacerbates renal insufficiency, atherosclerosis and glucose intolerance. Renal insufficiency was induced by 2/3 nephrectomy in LDL receptor knockout mice. Nx animals were given normal diet (Nx) or WD (Nx+WD). The controls were sham operated animals on normal diet (control) and WD (WD). To verify if LPS plays a role in exaggerating renal insufficiency, polymyxin (PM), a known LPS antagonist, and curcumin (CU), a compound known to ameliorate chronic kidney disease (CKD), was given to Nx animals on western diet (Nx+WD+PM and Nx+WD+CU, respectively). Compared to control, all other groups displayed increased circulatory LPS. The Nx+WD cohort had the highest levels of LPS. Nx group had significant renal insufficiency and glucose intolerance but not atherosclerosis. WD had intense atherosclerosis and glucose intolerance but it did not show signs of renal insufficiency. Compared to other groups, Nx+WD had significantly higher cytokine expression, macrophage infiltration in the kidney, renal insufficiency, glucose intolerance and atherosclerosis. PM treatment blunted the expression of cytokines, deterioration of renal function and associated disorders, albeit not to the levels of Nx, and was significantly inferior to CU. PM is a non-absorbable antibiotic with LPS binding properties, hence its beneficial effect can only be due to its effect within the GI tract. We conclude that LPS may not cause renal insufficiency but can exaggerate kidney failure and associated disorders following renal insufficiency.

## Introduction

Kidney failure is frequently associated with hypertension, glucose intolerance and cardiovascular disorders such as atherosclerosis. Inflammation, oxidative stress and dyslipidemia are the common mechanisms driving renal failure and associated disorders. The western style diet characterized by highly processed foods with high content of sugar and fat are the major contributor to type 2 diabetes, atherosclerosis and also increased incidence of chronic kidney disease [1]. Population based studies have determined that excess body weight was a strong independent risk factor for end stage renal disease (ESRD) even after adjustment for other major risk factors that are associated with ESRD [2]. However, the mechanisms by which obesity/metabolic syndrome due to high fat diet exacerbate renal failure remain elusive and largely speculative.

To understand this mechanism we investigated the effect of high fat high cholesterol diet, also known as western diet, on LDL receptor knockout (LDLR-/-) mice with renal failure. We have shown that western diet can produce atherosclerosis and glucose intolerance in LDLR-/- mice [3,4]. Others have shown that partial nephrectomized LDLR-/- mice on western diet have vascular calcification, osteodystrophy and hyperphosphatemia [5]. Apoe knockout mice, another mouse model of atherosclerosis, have accelerated atherogenesis following uremia [6,7]. The major distinction between Apoe knockout and LDLR-/- is their response to the diet. On a chow diet, ApoE knockout mice have high cholesterol (400 to 500 mg/dL) and atherosclerotic lesions whereas, the chow-fed LDLR-/- mice have mildly increased plasma cholesterol levels (175 to 225 mg/dL) and usually do not develop significant atherosclerotic lesions [8]. Since environmental factors such as diet govern the development of atherosclerosis and glucose intolerance in LDLR-/- mice, we theorized that the nephrectomized LDLR-/- mouse will be a suitable model to elucidate the mechanism by which western diet can influence hypertension, renal dysfunction, glucose intolerance and atherosclerosis.

In addition, consumption of western diet can cause mild endotoxemia [9] and studies in both mice and men, show that direct manipulation of the gut microbiome improves features associated with metabolic syndrome and obesity [10,11], indicating that gut microbes may be promising new targets for treating some diseases.

In this study we hypothesized that western diet will increase the intestinal para-cellular permeability of lipopolysaccharide (LPS) which will potentiate the inflammatory milieu already existing in renal failure due to the production of soluble uremic toxins resulting from bacterial metabolism of intestinal proteins [12]. We posit that aggravated inflammation will exaggerate renal dysfunction and its associated disorders. Furthermore, as a proof of concept, we show that the non-absorbable antibiotic polymyxin with LPS binding capability [13] significantly ameliorates inflammation and improves renal dysfunction and associated disorders. As a positive control we used curcumin which has significant anti-inflammatory properties with ameliorative effects on renal failure [14,15] and is reported to improve diabetes and atherosclerosis [16]. Furthermore, similar to polymixin, curcumin is a poorly absorbed compound [17] with antibacterial properties [16].

## Materials and Methods

Curcumin was purchased from Enzo Life Sciences (Farmingdale, NY). Polymixin was purchased from RPI (Prospect, IL). All antibodies were obtained from Santa Cruz Biotechnology (Santa Cruz, CA) unless otherwise stated.

### Animals

All animal procedures were approved by the Institutional Animal Care and Use Committees of Virginia Commonwealth University. The protocol number was AD-20190. LDLR-/- mice were divided into four groups with six animals in each group. The mice underwent either sham surgery or 2/3 nephrectomy by the procedure described below.

#### Plan of study

It has been shown that C57Bl/6 mice are not good models for CKD [18,19]. However, Bro et al [6][20] and Davis et al [5] have shown that Apoe-/- and LDLR-/- mice, both of which are of C57Bl/6 background are susceptible to uremia. Our goal was to understand if mild uremia can be aggravated by high fat diet and whether diet can also influence atherosclerosis and blood sugar which frequently accompanies renal failure.

#### Surgery

Renal failure was achieved by 2/3 nephrectomy where a one third of the right kidney was cut and after a week the left kidney was removed. The surgical procedure was similar to the 5/6^th^ nephrectomy done earlier in our laboratory in rats [15]. 8–10 weeks old, LDL receptor knock out (LDR-/-) mice from C57Bl/6 background were chosen. All operations were carried out in sterile conditions under isoflurane anesthesia. A left flank incision was made, and the left kidney was exposed. The upper one third portion of the left kidney was ligated and cut. The muscle and skin incisions were sutured with polypropylene suture. The animals were returned to the vivarium to recover. One week later, a right flank incision was made, the renal vessels and ureter were ligated to prevent blood flow, and the entire right kidney was excised. Thus these animals had 1 and 1/3^rd^ kidney removed by surgery. Animals were returned to the vivarium to recover, and treatment was started after 7 days. The animals were studied for 16 weeks and on the 17^th^ week they were sacrificed.

The animals were divided as follows:

#### Control

These are control animals who were sham operated and given normal chow.

#### WD

Western diet (TD-88137 from Harlan Teklad) is known to cause glucose intolerance and atherosclerosis in LDLR-/- mice. Severity of glucose intolerance and atherosclerosis in this strain of mice depended on the amount of diet consumed. After 8–10 days of the second surgery food consumption of the nephrectomized animals started to decrease. Since diet plays an important role in the development of atherosclerosis and glucose intolerance, the amount of western diet given to the animals was carefully monitored. The nephrectomized animals were given weighed amount of western diet at around 4 pm and allowed to eat ad lib. Next evening the diet was weighed and the average amount consumed was given to the sham operated animals and this group was designated WD. We had observed that the food consumption of the WD group was more than the nephrectomized group (Nx+WD and Nx; see below) therefore, we added normal chow to the WD group to prevent malnutrition. In the beginning we were concerned that the animals of WD cohort would avoid the western diet however, to our surprise the animals preferred the western diet over the normal chow. On an average the nephrectomized mice consumed three fourth of the control animals.

#### Nx

Nephrectomized on normal chow diet.

#### Nx+WD

Nephrectomized on high fat high cholesterol diet or western diet (TD 88137).

Treatment: Suspension of curcumin (100 mg/kg) was made in 0.5% carboxymethylcellulose (CMC). Curcumin is nonpolar and must be suspended in CMC. Due to the instability of curcumin in an aqueous system, the compound was made fresh and given by gavage within 10 min of the preparation.

Polymyxin was given in the drinking water at the concentration of 3.5 mg/ml, a dose which was used for gut sterilization [21,22].

Treated group was divided as follows:

#### Nx+WD

Nephrectomized mice on high fat high cholesterol diet (western diet -TD 88137).

#### Nx+WD+Gav

Nephrectomized mice on high fat high cholesterol diet (western diet-TD 88137) gavaged daily with 0.5% CMC.

#### Nx+WD+PM

Nephrectomized mice on western diet mice given polymixin daily.

#### Nx+WD+CU

Nephrectomized mice on western diet gavaged daily with curcumin.

### Serum urea nitrogen, serum creatinine and urinary albumin

Serum urea nitrogen (urea) and creatinine were measured by a Quantichrom creatinine™ and Quantichrom urea assay™ kit. (Bioassay system; Hayward, CA). Albumin was measured by mouse albumin ELISA assay kit (Bethyl Laboratories; Montgomery, TX).

### Lipopolysaccharide (LPS) measurement

Plasma LPS was analyzed by Lamilus Amebocyte Lysate (LAL) assay (Lonza, Walkersville, Maryland, USA) according to the manufacturer’s instructions, with the following modifications: samples were diluted 5-10-fold to avoid interference with background color and preheated to 70°C for 10 minutes prior to analyses.

### Longitudinal measurement of arterial pressure by tail plethysmography

Arterial pressure (BP) was determined by tail plethysmography as previously described [14] using the CODA 2 system (Kent Scientific, Torrington, CT). CODA 2 utilizes volume pressure recording sensor technology to measure rat tail blood pressure. This is a computerized, noninvasive tail-cuff acquisition system which can simultaneously measure systolic, diastolic, and mean arterial pressure without operator intervention. Before surgery, mice were trained for 3 days and were kept in a restraining holder for a 5- to 10-min period. On the fourth day, BP was recorded (*week 0*). During this period, 25 sequential readings were obtained. Readings within a range of 10 mmHg were averaged. Two weeks after the second surgery, the animals were retrained and BP was recorded every alternate week. In this study, we report the mean arterial BP (MAP) of each group.

### Histology

Animals from each group were studied for histological changes in the kidney. A portion of the kidney was cut and fixed in 10% buffered formalin for light microscopy. The basic scoring system, was described previously [14]. A minimum of 100 glomeruli were scored per animal by an observer blinded to the origin of the tissue. Sections were cut at 2-μm thickness and stained with periodic acid-Schiff.

### Intraperitoneal glucose tolerance tests (IPGTT)

IPGTT was done as described before [4]. Briefly LDLR−/− of different cohorts were fasted overnight and given a single bolus of glucose (2 mg/g body weight) intraperitoneally. Blood glucose levels were determined by commercially available glucometer using tail vein blood at 0 minutes (before ip injection), and 15, 30, 60, and 120 minutes after ip administration.

### Quantitative atherosclerosis analyses

Quantitative atherosclerotic enface analysis was as described before [23]. Briefly the aorta was dissected from the heart to the iliac bifurcation, cleaned of any surrounding tissue, opened longitudinally, pinned on black wax, and fixed for 24 h in 10% buffered formalin. The fixed aortas were imaged on the black background using a Canon digital camera fitted with a 60-mm, f/2.8-macro lens. Total area and area occupied by the lesions in the aortic arch and total aorta were determined using Axiovision Image Analysis software (Carl Zeiss). The person quantifying the area occupied by lesions was blinded to the identity of the images. Extreme care was taken to ensure that any residual adventitial fat that appeared translucent on the images was not included in the area occupied by the lesions that were dense and opaque.

### Homogenization

#### Kidney homogenates

Each kidney was cut and immediately frozen in liquid nitrogen and kept at −70°C until use. The frozen kidney was ground to a powder and then mixed in ice-cold RIPA buffer (Thermo Fisher Scientific, Rockford, IL) and protease and phosphatase inhibitors. The kidney was homogenized in an ice-chilled Dounce homogenizer at 4°C, and centrifuged at 4°C at 2500 rpm for 5 minutes. The supernatant was aliquoted and stored at −70°C until use.

### Immunoblotting

Kidney homogenates (75–100 μg total protein) were separated on a 4–20% SDS-PAGE gel, and proteins were transferred to a polyvinylidene difluoride membrane as described before [15]. After being briefly washed in phosphate-buffered saline containing 1% Tween 20 (PBS-T) and blocked in 5% nonfat dry milk, blots were incubated with appropriate antibodies in 5% nonfat dry milk overnight at 4°C. After being washed three to five times in PBS-Triton X-100, blots were subsequently incubated with secondary antibody appropriately diluted in 5% nonfat dry milk for 1 h at room temperature. After being washed three to five times in PBS, blots were developed using Lightning Chemiluminescence Reagent Plus and exposed to X-rays.

### Isolation of peritoneal macrophages

Thioglycollate-elicited peritoneal macrophages were harvested, and nonadherent cells were removed after 2 h, and medium was replaced with fresh growth medium [4]. For determination of gene expression, in some experiments, total RNA was isolated (using the RNeasy kit from Qiagen) from the adherent macrophages 24 h after plating.

### Quantitative real-time RT-PCR analysis

Total RNA was extracted from kidneys with the RNeasy Mini Kit as described before [15]. Briefly, 2 μg of total RNA were reverse transcribed with the Thermoscript RT-PCR System (Invitrogen), and first-strand cDNA was used to perform real-time PCR using the Stratagene Mx3000p real-time PCR system with TaqMan Universal PCR Master Mix and optimized probe and primer sets from Applied Biosystems (Foster City, CA). The following probes were used: CD36 Mm00432403_m1), SRA (Mm00446214_m1). The amount of mRNA was calculated by the ΔΔCT method and normalized to β-actin.

### TNFα and IL6 ELISA

Cytokines were measured in kidney homogenates as described Wu et. al [24] with slight modification. ELISA kits for IL-6 and TNFα were obtained from BD Biosciences (San Jose, CA).

### Statistical analysis

Statistical comparisons among groups were performed using one way and two way ANOVA. Longitudinal measurements followed over time are analyzed using repeated measures of ANOVA. Groups were considered to be significantly different if p ≤ 0.05.

## Results

Obesity has been shown to be a risk factor for the development of kidney disease [25] but at the same time has been proven to be beneficial in various stages of chronic kidney disease [26,27]. However, the role of over-nutrition and the quality of nutrition has not been clearly elucidated in renal failure. Therefore, we investigated the effect of high fat high cholesterol or western diet on renal failure induced by 2/3 nephrectomy of LDLR-/- mice.

### 2/3^rd^ Nephrectomy and western diet does not affect kidney histology

In our earlier studies with 5/6^th^, nephrectomized rats we had seen global and segmental sclerosis with tubular dilation and atrophy [14,15]. In these 2/3^rd^. nephrectomized LDLR-/- mice we saw only protein droplets. There was no significant sclerosis or tubular damage (data not shown). However, as described below, serum creatinine and urea were significantly elevated in the 2/3^rd^ nephrectomized animals suggesting that the animals had renal i without significant histological abnormalities suggesting early stages of CKD.

### Western diet aggravates renal failure in the 2/3^rd^ nephrectomized (Nx) LDLR-/- mice

It is to be noted that anorexia and loss in body weight is frequently observed in CKD patients and animal models [15,27]. Changes in body weight following high fat/ western diet is shown in Table 1. The decrease in body weight in Nx animals was offset when western diet was given to nephrectomized animals. The WD group did not show significant changes in urea and creatinine (Table 1). However, as judged by urea and creatinine levels, high fat diet significantly aggravated renal failure (Table 1). Two way ANOVA confirmed that diet cannot induce renal failure per se, but confirmed that if renal function is compensated western diet can influence the biomarkers of renal failure.

**Table 1:** Changes in body weight, serum urea and creatinine in sham operated LDLR-/- mice fed on normal diet (control), sham operated mice fed on western diet (WD), 5/8 nephrectomized mice fed on normal diet (Nx) and 5/8 nephrectomized mice fed on western diet (Nx+WD). Statistically significant difference between the groups are shown in the comments.

	Control	WD	Nx	NX+WD	Comments
Body weight (gms)	27.08±1.76N = 6	35.42±4.50N = 5	19.6±2.0N = 5	27.52±5.36N = 5	Control vs WDP < 0.01Control vs NxP< 0.05 WD vs NxP < 0.001 WD vs NX+WDP < 0.01 Nx vs NX+WDP < 0.05
Serum Urea(mg/dl)	18.9±2.83N = 5	19.2±1.50N = 4	39.0±6.16N = 5	58.6±15.86N = 5	Control vs Nx+WDP < 0.001WD vs Nx+WDP < 0.001
Serum Creatinine(mg/dl)	0.45±0.08N = 5	0.43±0.07N = 5	0.7±0.05N = 5	0.92±0.09N = 5	Control vs NxP < 0.001Control vs Nx+WDP < 0.001WD vs NxP < 0.001WD vs Nx+WDP < 0.001Nx vs Nx+WDP < 0.001

### Changes in metabolic parameters

**Fig 1:**
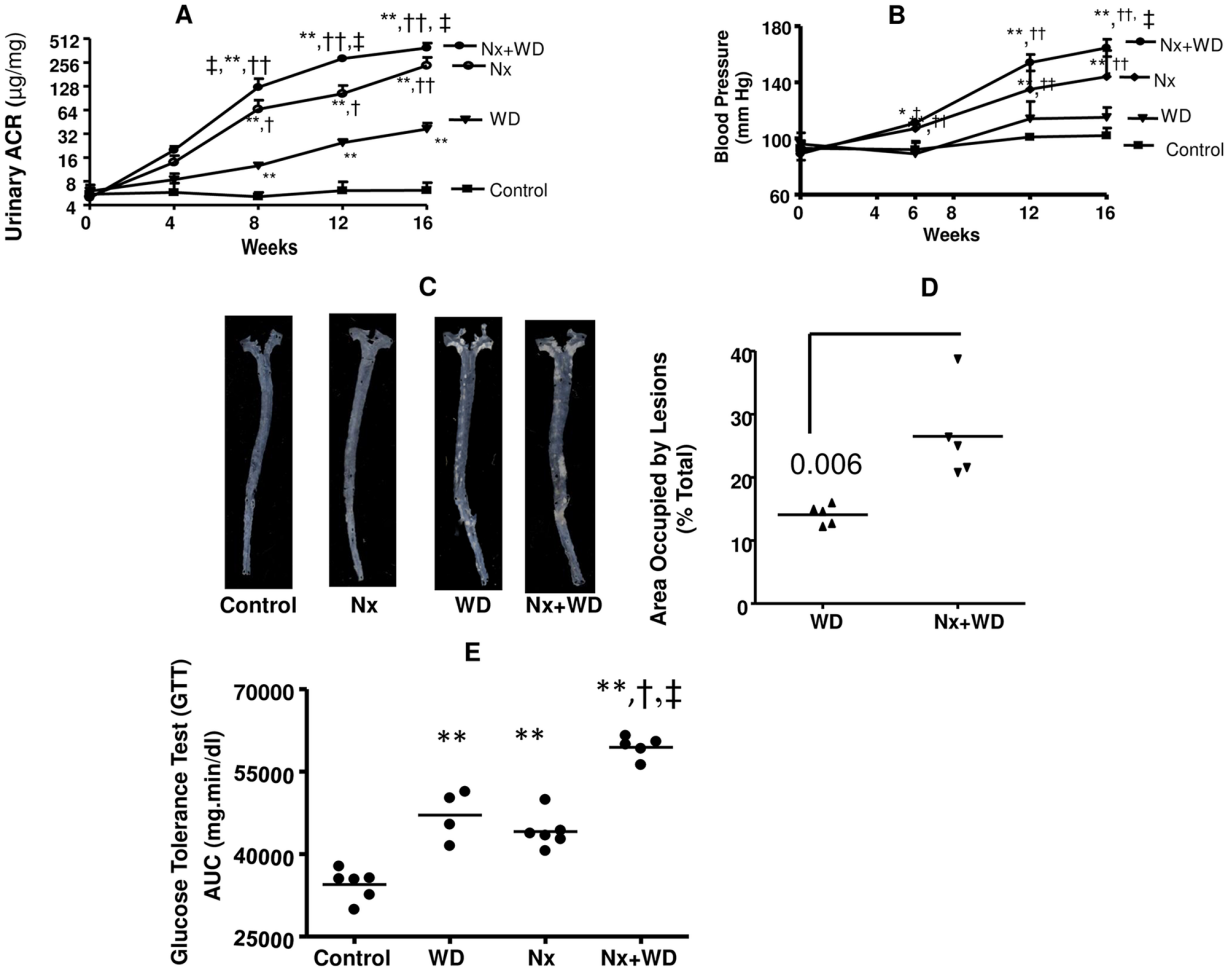
Western diet aggravates metabolic dysfunction in 5/8^th^ nephrectomized LDLR-/- mice. The four groups of LDLR-/- mice were divided as follows: sham operated (control), sham operated mice pair fed on western diet (WD), nephrectomized mice on normal diet (Nx), and nephrectomized mice on western diet (Nx+WD). Panel A shows the longitudinal changes in urinary albumin creatinine ratio. Panel B shows the longitudinal changes in blood pressure of the different groups. Panel C is the enface picture of atherosclerotic lesions after 16 weeks study. Panel D is the quantification of atherosclerotic lesions. Control and Nx did not show any significant atherosclerosis. Panel E is the AUC values of glucose tolerance tests (GTT) following ip injection of 2 mg/g glucose after 16 weeks of study. The values are means ± SD. The significant changes are represented by the following signs, *p<0.05 from control; **p<0.1 from control; †p<0.05 from WD, ††p<0.01 from WD; #p<0.05 from Nx+WD, ##p<0.01 from Nx+WD; ‡p<0.05 from Nx.

It has been recently shown that western diet can cause lipid accumulation in the kidney and an increase in the urinary albumin creatinine ratio (ACR) of C57Bl6 mice [28]. With the exception of control, the urinary ACR of all the groups significantly increased with time (Fig 1A). Repeated measure ANOVA showed that after 8 hours the urinary ACR of each time point was significantly higher than the previous time point. As shown in Fig 1B, by and of itself western diet did not have any effect on blood pressure but elevated the blood pressure in nephrectomized animals. After repeated measure two way ANOVA we conclude that diet significantly affected ACR and BP in animals with renal failure. The WD animals had significant atherosclerosis. Enface analysis of the aorta of control and nephrectomized animals revealed no significant atherosclerotic lesions (Fig 1C). However, nephrectomized animals on western diet had two fold higher atherosclerotic lesions than the WD group (Fig 1C and 1D). Glucose tolerance tests (Fig 1E) showed that the area under the curve of blood (AUC) glucose versus time was significantly elevated for both WD and nephrectomized animals. However, the highest AUC was observed in the Nx+WD group, suggesting that western diet can adversely influence diabetes in renal failure.

### Circulatory LPS and macrophage influx in the kidney is increased with renal failure and further exaggerated with western diet

It is well known that LPS is a potent inflammatory molecule which originates from the Gram-negative bacteria. LPS comes out in the circulation due to leaky gut phenomenon seen in CKD patients. Furthermore this can significantly increase the influx of macrophages in the kidney. As seen in Table 2, LPS levels in animals of the nephrectomized western diet group (Nx) were significantly higher than control. However, compared to Nx+WD the LPS levels of Nx and WD were two fold lower. This suggests that diet and renal failure can influence the permeability of LPS into the circulation. The macrophage marker CD 68 and SR-1 levels in kidney, in all groups, were significantly higher than the control. The highest levels were seen in Nx+WD cohort suggesting western diet can influence macrophage migration in the kidney and this can be exaggerated in renal failure.

**Table 2:** Changes in serum lipopolysaccharide (LPS) and macrophage markers CD68 and SR 1 in the kidney (measured by qPCR) in sham operated in LDLR-/- mice fed on normal diet (control), sham operated mice fed on western diet (WD), 5/6 nephrectomized mice fed on normal diet (Nx) and 5/6 nephrectomized mice fed on western diet (Nx+WD). Statistically significant difference between the groups are shown in the comments.

	Control	WD	Nx	NX+WD	Comments
Serum LPS(Endotoxin unit/ml)	0.444±0.061N = 5	1.57±0.31N = 4	1.97±0.82N = 5	4.13±0.74N-5	Control vs WDP < 0.05Control vs NxP < 0.01Control vs Nx+WD P < 0.001WD vs NX+WDP < 0.001 Nx vs NX+WDP < 0.001
CD 68(% Control)	100.0±6.91N = 5	231.3±34.77N = 4	342.1±38.85N = 5	504.0±62.15N-5	Control vs WDP < 0.01Control vs NxP < 0.001Control vs Nx+WDP < 0.001WD vs NxP < 0.01WD vs Nx+WDP < 0.001Nx vs Nx+WDP < 0.001
SR-1(% Control)	100.0±8.46N = 5	158.8±4.86N = 4	321.5±26.35N = 5	449.5±22.99N = 5	Control vs WDP < 0.01Control vs NxP < 0.001Control vs Nx+WDP < 0.001WD vs NxP < 0.01WD vs Nx+WDP < 0.001Nx vs Nx+WDP < 0.001

**Fig 2:**
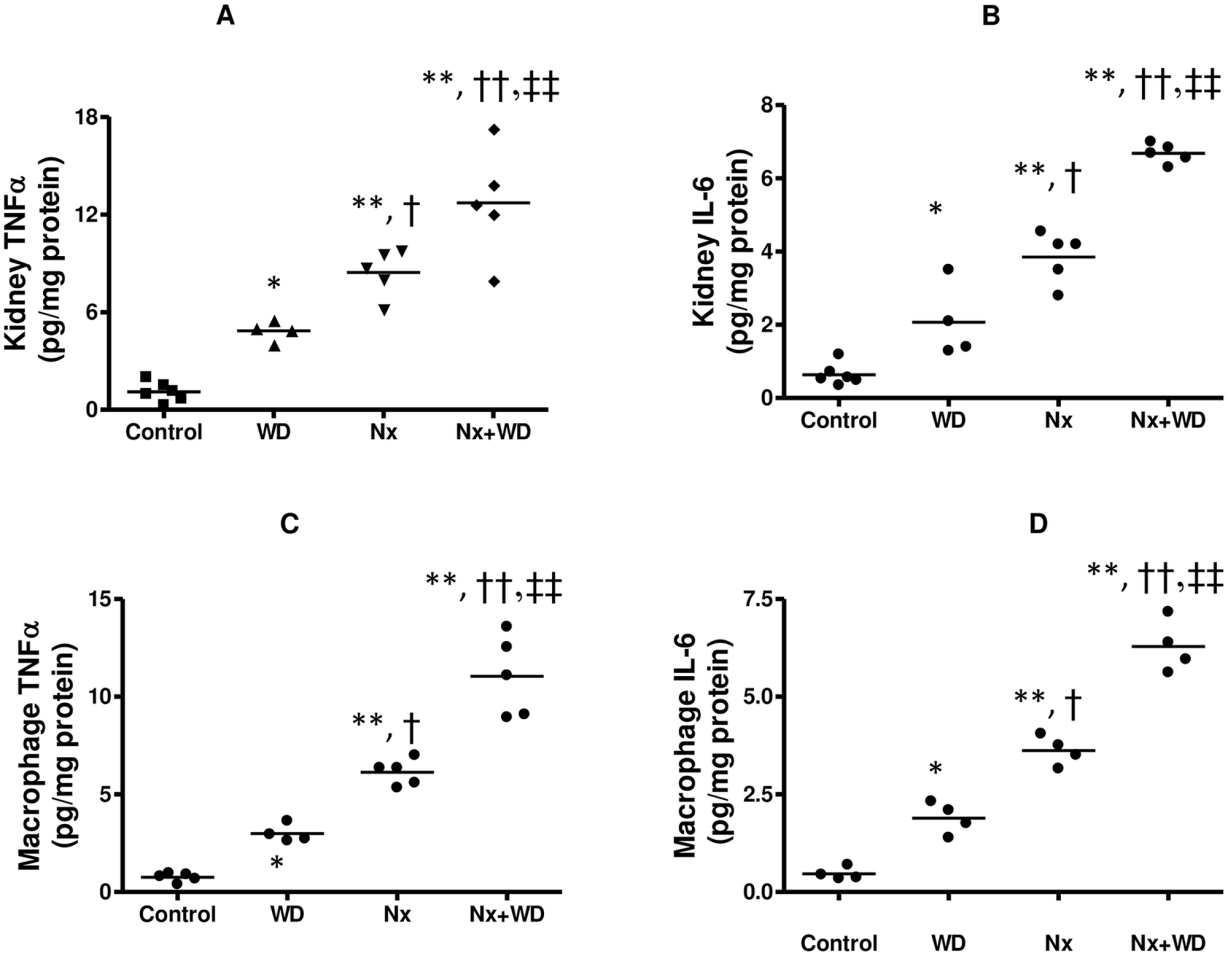
Kidney and macrophage TNFα and IL6. The four groups of LDLR-/- mice were divided as follows: sham operated (control), sham operated mice pair fed on western diet (WD), nephrectomized mice on normal diet (Nx), and nephrectomized mice on western diet (Nx+WD). At the end of 16 weeks the kidney TNFα (Fig 2A) and IL6 (Fig 2B) were measured by ELISA. Thioglychollate elicited macrophages were plated as described in the methods and TNFα (Fig 2C) and IL6 (Fig 2D) were measured by ELISA. The values are means ± SD. The significant changes are represented by the following signs *p<0.05 from control; **p<0.1 from control; †p<0.05 from WD, ††p<0.01 from WD; #p<0.05 from Nx+WD, ##p<0.01 from Nx+WD; ‡p<0.05 from Nx, ‡‡ p<0.01 from Nx.

We also looked at the cytokine expression in the kidney and thioglycollate-elicited peritoneal macrophages from these four groups of mice. As seen in Fig 2, the changes in cytokine expression in both the kidney and macrophages were qualitatively similar. Our data demonstrate that the nephrectomized mice had a significant increase in TNFα and IL-6 in both kidney and macrophages. Furthermore, as illustrated by two way ANOVA, diet significantly influenced cytokine expression in both kidney and macrophages of animals with renal failure. This suggests that continuous feeding of western diet can aggravate inflammation. The increased influx of macrophages can contribute to the increase in kidney cytokine levels however, in this study we have not determined the extent of macrophage’s contribution to the kidney cytokine expression.

### Rationale for using polymyxin and curcumin

High fat diet, by disrupting the intestinal barrier, can increase LPS absorption in both men and mice [9,29]. Circulating LPS has been observed in patients with renal failure [30,31] which may be due to the breach in in the intestinal barrier seen in CKD [32]. We speculated that if we decrease LPS levels in the circulation or improve intestinal barrier function we might ameliorate renal failure. Polymixin, a non-absorbable antibiotic that can bind and neutralize endotoxins [13], was used to understand if LPS binding helps in renal failure. Curcumin was used as a positive control because it is known to ameliorate renal failure [14,15] and, in addition, it is known to correct intestinal barrier function [33]. Since the nephrectomized animals on western diet (Nx+WD) had the worst renal outcome, we chose this group to study the effect of polymixin and curcumin.

### Effect of polymixin and curcumin on renal function

Body weight was not significantly affected the treatment. Curcumin decreased both creatinine and urea (Table 3). Although, polymyxin treatment improved urea levels it did not alter creatinine (Table 3).

**Table 3:** Effect of Polymyxin and curcumin on body weight, serum urea, and creatinine of 5/6 nephrectomized mice fed on western diet (Nx+WD). Nx+WD rats were treated with polymixin (Nx+WD+PM) and curcumin (Nx+WD+CU). PM was dissolved in drinking water and freshly prepared CU was given orally everyday by gavage the respective control (Nx+WD+Gavage) received daily gavage of the vehicle. The data shown is the mean and SD of 5 rats per group. Statistically significant difference between the groups are shown in the comments.

	NX+WD	NX+WD+PM	NX+WD+Gavage	NX+WD+CU	Comments
Body weight (gms)	27.52±5.36	25.84±5.20	27.80±4.10	25.60±3.74	
Serum Urea(mg/dl)	58.6±15.86	25.95 ±7.96	59.57±13.32	18.61 ±6.205	Nx+WD vs Nx+WD+PM P < 0.01Nx+WD+Gav vs Nx+WD+CUP< 0.001
Serum Creatinine(mg/dl)	0.92±0.09	0.75±0.13	0.98±0.05	0.71±0.07	Nx+WD+Gav vs Nx+WD+CU P < 0.05Nx+WD+PM vs Nx+WD+CUP < 0.05

### Effect of polymyxin and curcumin on metabolic parameters

**Fig 3:**
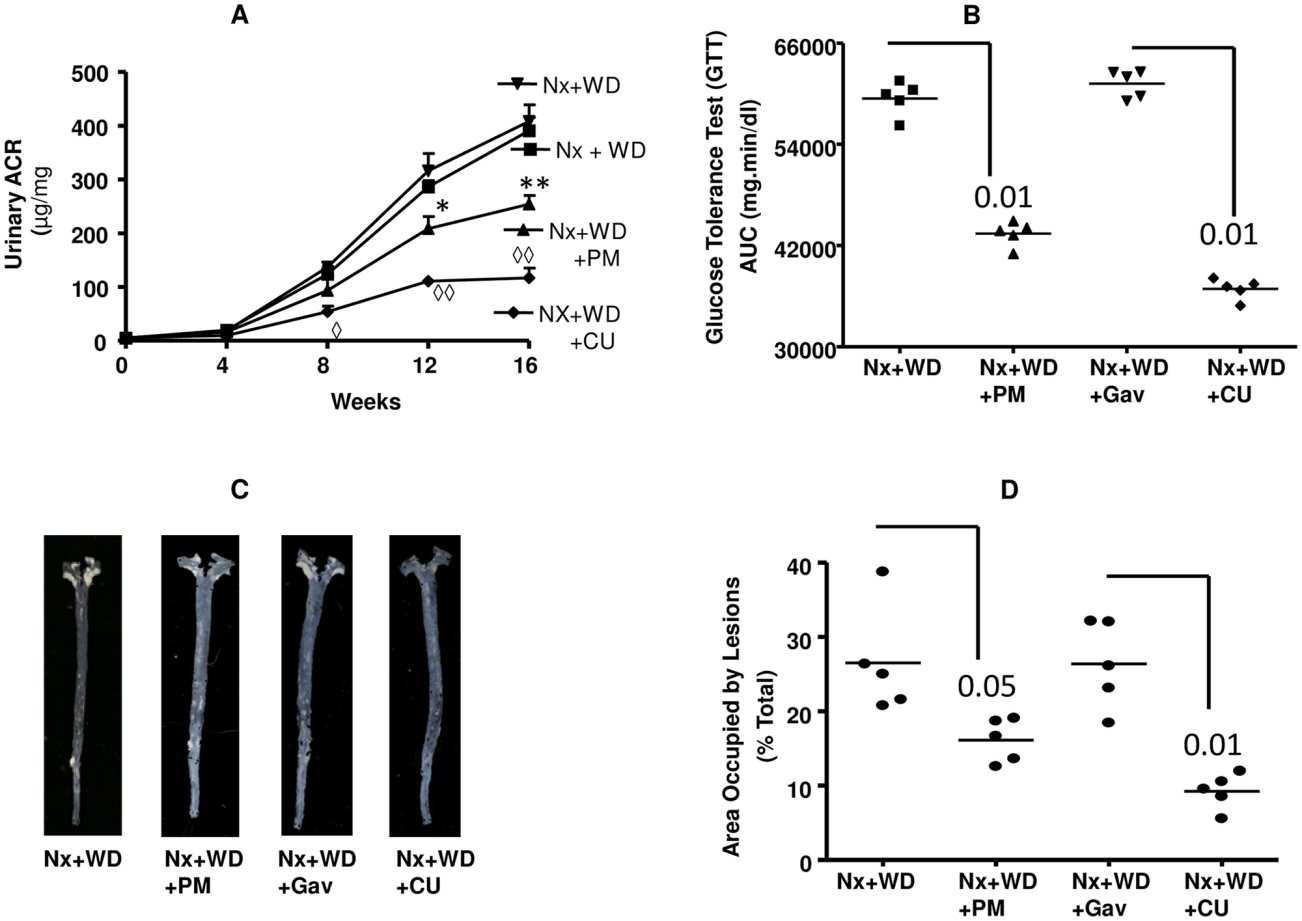
Effect of polymixin (PM) and curcumin (CU) on metabolic functions. Nephrectomized animals on western diet (Nx+WD) were treated with polymixin in drinking water (Nx+WD+PM) and curcumin gavage (Nx+WD+CU) for 16 weeks. The control for Nx+WD+Cu was gavaged with vehicle for 16 weeks (Nx+WD+Gav). Panel A, shows the longitudinal changes in urinary albumin creatinine ratio. Panel B is the AUC values of glucose tolerance tests (GTT) following ip injection of 2 mg/g glucose after 16 weeks of study. Panel C is the enface picture of atherosclerotic lesions after 16 weeks study. Panel D is the quantification of atherosclerotic lesions. * p<0.05 compared to Nx+WD; ** p<0.01 compared to Nx+WD, ◊ p<0.05 compared to Nx+WD+Gav, ◊◊ p<0.01 compared to Nx+WD+Gav.

Neither polymyxin nor curcumin had any effect on blood pressure (data not shown). Curcumin treated animals showed a significant decrease in urinary ACR after 8 weeks (Fig 3A). The decrease in ACR was evident in the PM group from 12 to 16 weeks (Fig 3A). As shown in Fig 3B, both polymyxin and curcumin significantly improved the glucose tolerance test of nephrectomized animals on high fat diet by 1.4 fold and 1.7 fold respectively (p<0.01). Fig 3C shows the enface picture of the aorta from nephrectomized animals on high fat diet which are compared with curcumin and polymyxin treated animals. As shown in Fig 3D curcumin reduced the lesion area by 63% (P<0.01) and polymyxin reduced the area by 33% (p<0.05).

### Effect of polymyxin and curcumin on circulatory LPS and macrophage influx in the kidney

**Fig 4:**
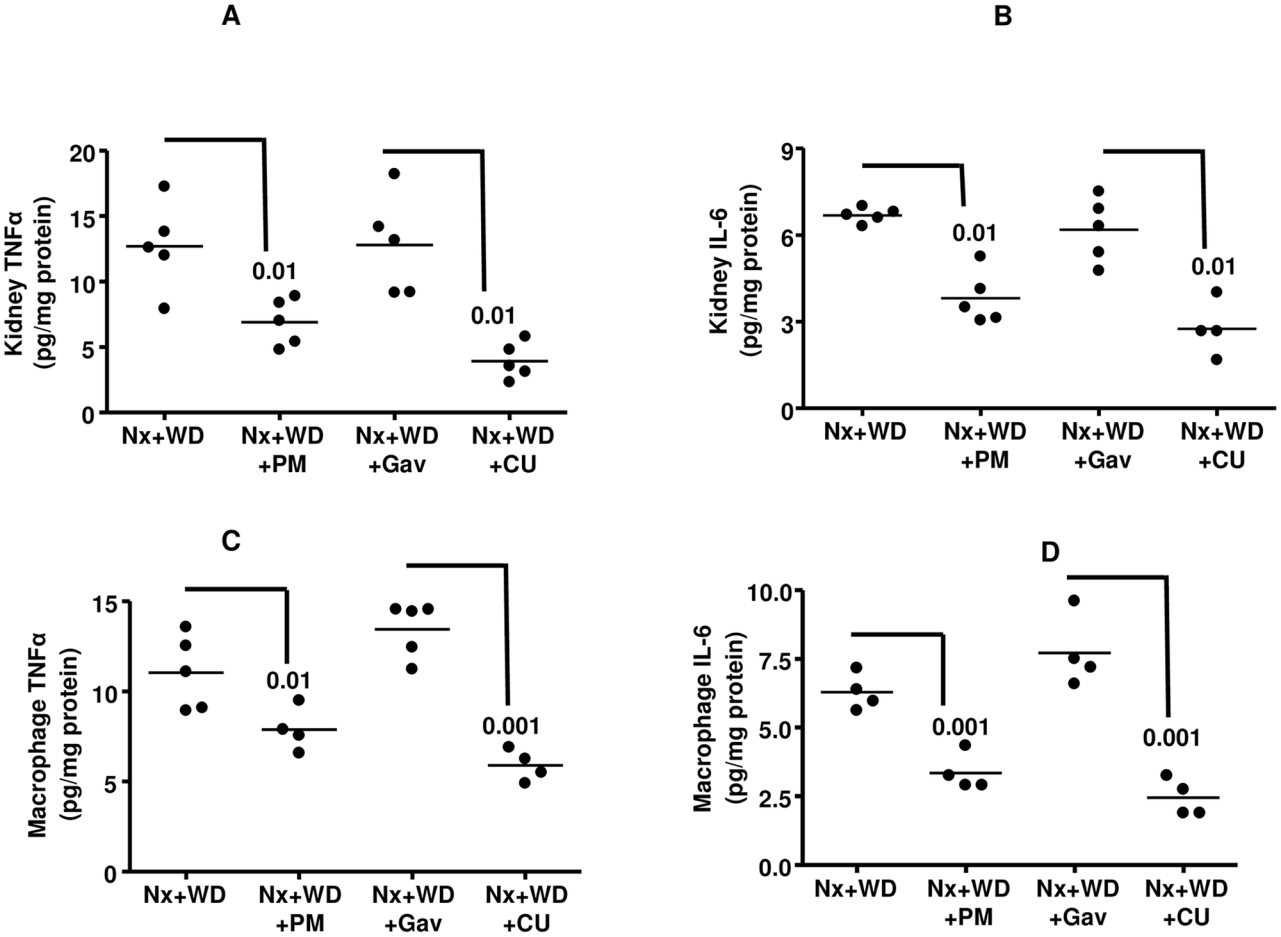
Effect of polymixin (PM) and curcumin (CU) on kidney and macrophage cytokine expression. Nephrectomized animals on western diet (Nx+WD) were treated with polymixin in drinking water (Nx+WD+PM) and curcumin gavage (Nx+WD+CU) for 16 weeks. The control for Nx+WD+Cu was gavaged with vehicle for 16 weeks (Nx+WD+Gav). The kidney TNFα and IL-6 are shown in panels A and B respectively. The expression of TNFα and IL-6 from thioglychollate elicited macrophages are shown in panels C and D. The expression of cytokines in both kidney and macrophages were analyzed by ELISA.

Both polymyxin and curcumin significantly reduced circulatory LPS levels (p<0.001). The reduction by polymyxin was 63%, whereas curcumin reduced LPS level by 36% (Table 4). The influx of macrophages in the kidney, as measured by the presence of macrophage markers CD 68 and SR-1, was significantly reduced by both polymyxin and curcumin (Table 4). The cytokine expression in the kidney and thioglycollate-elicited peritoneal macrophages were significantly reduced by both polymyxin and curcumin (Fig 4). As shown in Fig 4A, polymyxin and curcumin reduced kidney TNFα by 46% (p<0.01) and 69% (p<0.01) respectively. Kidney IL6 was reduced by polymyxin and curcumin by 43% (p<0.01) and 55% (p<0.001) respectively (Fig 4B). Macrophage TNFα was reduced by polymyxin and curcumin by 47% (p<0.01) and 42% (p<0.01) respectively (Fig 4C). The macrophage IL6 concentration was reduced 57% (p<0.001) by polymyxin and 61% (p<0.001) by curcumin (Fig 4D).

**Table 4:** Polymixin (PM) and curcumin (CU) favorably modulates serum LPS levels and macrophage influx in the kidney of the 5/6 nephrectomized animals on western diet (Nx+WD). The macrophage markers CD68 and SR-1 were quantitated by qPCR as described in the method. Polymyxin was given in drinking water (Nx+WD+PM) and the data compared with Nx+WD cohort. Freshly prepared curcumin was given daily by gavage to nephrectomized animals on western diet (Nx+WD+CU) and the results are compared with its control, nephrectomized animals on western diet gavaged daily with vehicle (Nx+WD+Gav). Statistically significant difference between the groups are shown in the comments.

	NX+WD	NX+WD±PM	NX+WD±Gav	NX+WD±CU	Comments
Serum LPS (Endotoxin unit/ml)	4.13±0.74N = 5	1.52±0.31N = 4	4.34±0.31N = 5	2.72±0.59N-4	Nx+WD vs Nx+WD+PMP < 0.001Nx+WD+PM vsNx+WD+GavP < 0.001
CD 68 (% Control)	100.0±21.18N = 5	61.93±13.18N = 5	100.0±11.91N = 5	53.76±6.06N-5	Nx+WD vs Nx+WD+PMP < 0.001Nx+WD+PM vs Nx+WD+GavP < 0.001
SR-1(% Control)	100.0±7.23N = 5	66.08±7.46N = 5	100.0±6.76N = 5	56.89±6.76N = 5	Nx+WD vs Nx+WD+PMP < 0.001Nx+WD+PM vs Nx+WD+GavP < 0.001

## Discussion

These results show that polymixin reduces BUN and ACR levels but not creatinine suggesting it may have some beneficial effect on kidney function. It also improves atherosclerosis and IPGTT, albeit less efficiently than curcumin. It can also decrease macrophage influx and cytokine expression. To the best of our knowledge polymyxin is a non-absorbable antibiotic with a LPS binding property. Therefore it cannot enter the systemic circulation. Hence we speculate that its beneficial effect is imparted by modulating LPS permeability from the gut.

Furthermore, these studies demonstrate that consumption of western diet exacerbate renal dysfunction induced by 2/3^rd^ nephrectomy. Western diet aggravates the disruption of intestinal barrier function in renal failure, further exaggerating the release of intestinal LPS into the systemic circulation, leading to increased inflammation. Partial nephrectomy of LDLR-/- mice caused a significant increase in serum creatinine, BUN, and ACR which were further elevated in nephrectomized mice on western diet. However, there were no significant histological changes in the kidney from which it can be surmised that these animals were in renal failure equivalent to the early stages of CKD. One way ANOVA analysis clearly showed that western diet did not affect creatinine and urea in non-nephrectomized animals but greatly enhanced renal failure in the animals whose renal function was compromised. Further analysis with two way ANOVA confirmed that diet, per se, cannot affect renal function but can modulate renal failure following nephrectomy.

Renal failure is frequently accompanied with hypertension, and in this mouse model of renal failure we found significantly elevated BP. ACR has significant predictive value in renal failure, cardiovascular disease, and diabetes [34]. All experimental groups had significantly high ACR. However, WD group had normal renal function. We speculate that the changes in urinary ACR in these mice are probably predictive of glucose intolerance and atherosclerosis rather than deteriorating renal function. The ACR of the Nx+WD group was significantly higher than the rest of the cohorts. This group also had the worst renal outcome. This is similar in humans where it is known that in type 2 diabetes, albuminuria is a strong predictor of declining end stage renal disease [35] and progression of prediabetes to diabetes [36]. The uremic animals on normal diet had significant weight loss which was offset by western diet. However, as seen from two way ANOVA analysis, diet does not contribute or have significant interaction with nephrectomy i.e. renal failure. Pro-inflammatory cytokines, released during chronic renal failure, act on the central nervous system to alter the release and function of several key neurotransmitters, thereby altering both appetite and metabolic rate [37]. We postulate that the change in the body weight observed in our study is the result of renal failure and altering the body weight does not affect the outcome of renal function.

High fat, high energy diet alters intestinal paracellular permeability leading to an increase in LPS levels in plasma [9,29] and this is related to the changes in gut microbiota [30,38–40]. One of the limitations in this study is that we did not look into the role of gut microbiota. More extensive studies are required to understand the relationship between gut microbiota and LPS secretion, since gut microbiota are different in these species [41]. Vaziri and colleagues have shown that uremia can deplete colonic tight junction proteins leading to the disintegration of colonic epithelium [39]. The increase in the circulatory LPS following renal failure and the exacerbation of endotoxin levels following western diet are possibly due to increased intestinal paracellular permeability. A similar increase in circulating endotoxins has also been reported in patients with renal failure [31,42]. One should be cognizant of the fact that the amount of circulatory endotoxin seen in CKD patients or nephrectomized animals is significantly less robust than seen in certain inflammatory conditions such as sepsis where inflammation is robust enough to cause mortality. However, even with low concentrations of LPS, there can be an alteration of macrophage cytokine production which will affect inflammation. In fact, it has been shown that pretreatment with a high dose of LPS followed by retreatment with LPS, profoundly inhibited TNFα secretion, a phenomenon known as endotoxin tolerance [43]. In contrast, very low dose LPS pretreatment significantly augmented TNFα and IL-6 production when they were restimulated with various concentration of LPS [44–47]. Furthermore, it has been observed that mice primed with low dose LPS *in vivo* experience significantly elevated mortality following a second hit of high dose LPS [47]. These studies clearly indicate that low levels of LPS, such as those seen in renal failure, can aggravate inflammation by increasing cytokine production. Dietary saturated fatty acids such as palmitic acid, have been implicated in promoting inflammation, metabolic syndrome and atherosclerotic cardiovascular disease [48]. Toll like receptor 4 (TLR4) has been shown to play an important role in diabetes and cardiovascular disease [49], and both fatty acids and LPS are ligands for TLR4 receptors [48,50]. It has been shown that low levels of LPS not only increase cytokine production but also prime macrophages and produce exaggerated inflammation if they are re-exposed to LPS [44–46]. Moreover, LPS induced priming effects are not restricted to the LPS stimulus alone, but also extend to other stimuli [51]. Therefore, it is possible that low dose LPS may also prime macropahges to fatty acid mediated inflammation. We have shown that low dose LPS augments fatty acid mediated activation of pro-inflammatory cytokines and NFkB [33]. This signifies that small levels of circulating LPS from renal failure can markedly augment the inflammatory potential of fatty acids from western diet. Furthermore, it has been shown that TLR4 signaling can promote innate immune cell migration [52]. In this study we have seen a significant increase in macrophage markers in the kidney which was accompanied by increased cytokine levels in the kidney. These cytokines could be generated by the infiltrated macrophages and/or from the kidney cells. Either way, it will lead to exacerbation of inflammation.

Curcumin treatment is known to ameliorate renal failure [14,15], type 1 diabetes [53] and atherosclerosis [54]. Antioxidant, anti-inflammatory and various other mechanisms have been attributed as a mechanism for the efficacy of curcumin in combating all the disease process [55]. However, it must be noted that curcumin has very poor bioavailability [17] suggesting that a considerable effect of this compound might be mediated in the intestine. We observed that curcumin significantly reduced LPS levels in the circulation of the Nx+WD animals, suggesting it may play a role in modulating intestinal permeability. Based on the changes in plasma LPS we have emphasized that the inflammation is due to LPS secreted from the gut. It is to be noted that curcumin and polymyxin can alter the biochemical milieu of the intestinal tract and, as such, may alter the structure, composition, and function of microbial flora which can result in the change in secretion of other inflammatory toxins such as oxalate, and uric acid [38]. We have concentrated on LPS because in a recent study we have shown that LPS can disrupt the barrier function of intestinal epithelial cells (Caco-2 cells) by down regulating intestinal tight junction proteins ZO-1 and claudin-1 expression [33]. Furthermore, curcumin can restore the intestinal barrier function not only by upregulating the tight junction proteins but also by increasing intestinal alkaline phosphatase activity which can dephosphorylate LPS rendering it inactive [33]. All these explanations lead us to the conjecture that LPS play a major role in renal failure however, further studies using TLR4 knockout mice will be required to determine the exact role of LPS in renal dysfunction. In this study, we have shown curcumin can significantly reduce TNFα. In our earlier study, we have shown it also reduces IL-1β [15]. Both these cytokines (TNFα and IL-1β) are shown to increase intestinal paracellular permeability [56,57]. Curcumin has significant antibacterial activity [58], poor bioavailability [17] and reduces LPS absorption. These facts, in association with its effect on the cytokines, lead us to speculate that some of its beneficial action may be mediated by affecting gut permeability.

Polymixin, a non-absorbable antibiotic with LPS binding capabilities [13], was introduced in the treatment regimen as proof of concept that neutralization of LPS and by reducing leakage of LPS into the circulation can reduce inflammation, and thereby improve renal function and associated disorders. As seen in this study, polymyxin significantly reduced macrophage infiltration in the kidney, decreased cytokine levels, and improved glucose tolerance and atherosclerosis. Although it reduced serum urea and ACR, it did not significantly reduce creatinine. The exact reason for this discrepancy is not clear. However, it may be due to incomplete neutralization of LPS. Polymyxin significantly reduced LPS levels but did not take it down to the levels of control group suggesting it may not be efficacious in the treatment of renal failure. It can be argued that the LPS lowering effect of curcumin was inferior to polymixin. But, unlike polymyxin, it significantly reduced creatinine, and was superior in positively affecting many of the biomarkers of inflammation, renal failure, glucose intolerance and atherosclerosis. In light of this argument, it needs to be stated that curcumin has significant anti-inflammatory and anti-oxidant properties which may have resulted in a superior pharmacological effect. In spite of poor absorption, curcumin reaches the circulation and both curcumin and its metabolites (which can also be generated by the intestinal epithelium [59,60] have significant anti-inflammatory and antioxidant properties [55]. These will positively influence renal failure and associated disorders. In contrast, the pharmacological effect of polymyxin is limited to its bactericidal and LPS neutralizing/binding properties [13]. Although this explains the inferiority of polymixin it also proves that the effectiveness of polymyxin in this study is related to LPS binding, decreased absorption of LPS and possibly altering the gut microbiota. Furthermore, it provides evidence that LPS plays a significant role in modulating renal dysfunction, glucose intolerance and atherosclerosis. We further conclude that western diet increases paracellular permeability of LPS and, along with palmitic acid, can aggravate renal failure and associated disorders.
